# Discal Cyst of the Lumbar Spine: Case Report of a Rare Clinical Entity

**DOI:** 10.5704/MOJ.1807.011

**Published:** 2018-07

**Authors:** R Sanjeevan, S Prabu, A Azizul, Y Abdul-Halim

**Affiliations:** Department of Orthopaedics, Universiti Sains Malaysia, Kubang Kerian, Malaysia

**Keywords:** discal cyst, endoscopic excision, intraspinal extradural cysts

## Abstract

Being a rare clinical entity, discal cyst presents indistinguishably from other causes of lower back pain and radiculopathy. It is an extremely rare pathology with unclear pathogenesis, indeterminate natural history with no consensus on the ideal management of the condition. We report a rare case of discal cyst in a patient who presented to our centre with localised low back pain and subsequently left sided radicular pain. With the aid of MRI and with clear surgical indication we proceeded with endoscopic removal of the cyst and intraoperatively confirmed its origin from the adjacent disc. The patient had immediate relief of his symptoms and no postoperative complications. We recommend that endoscopic surgery can be an effective alternative to conventional open surgery for discal cyst of the lumbar spine.

## Introduction

Discal cysts are defined as intraspinal extradural cysts with a distinct communication with the corresponding intervertebral discs^1^,^2^. Younger male are more susceptible to discal cysts compared to degenerative lumbar disc herniation^3^. Discal cyst is one of the differential diagnoses of an intraspinal extradural cystic lesion^4^. MRI has facilitated the diagnosis in a non-invasive manner contrary to earlier reports recommending discography as a diagnostic tool. Development of an accurate understanding regarding the treatment of the discs remains quite challenging due to limited literature and research in this topic.

## Case Report

**Fig. 1:**
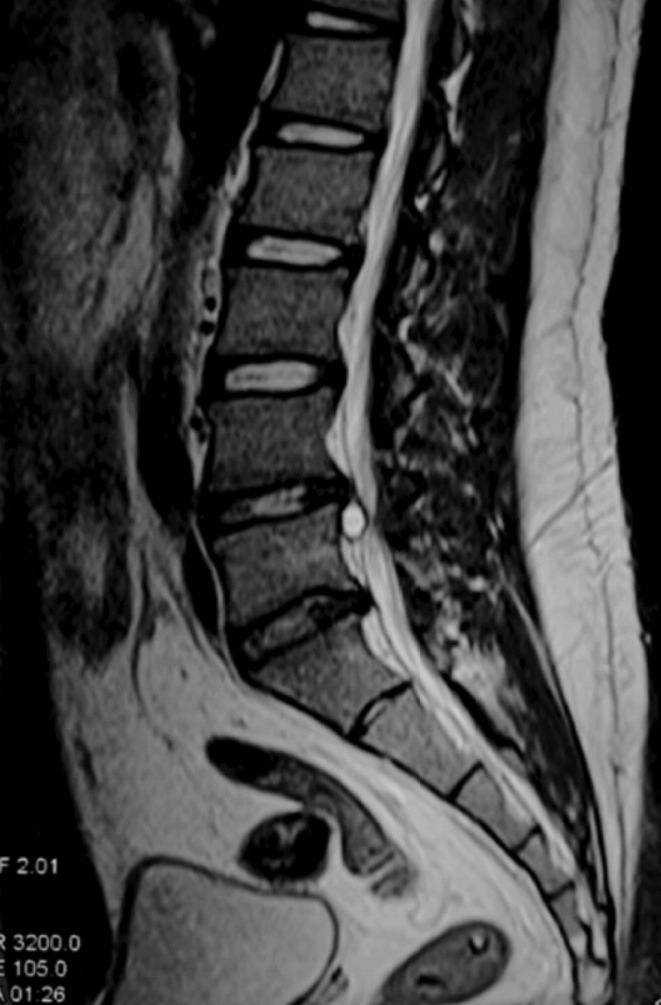
Sagittal view of the lumbar spine MRI showing a cystic lesion in the anterior epidural space with high signal intensity on T2-weighted image.

We present the case of a 23-year old male with the chief complaint of mechanical low back pain of eight months duration. He gave a history of having fallen in the sitting position on two different occasions during his martial arts practice. Initially, his symptoms were localised, infrequent and aggravated by prolonged sitting. Subsequently, after six months, he developed sciatica over his left lower limb radiating distally to the dorsum of his foot and associated with numbness. There was no weakness or any other ominous signs. Systemic review was unremarkable and he had no constitutional symptoms. The left straight leg raising test was positive at 60 degrees. Neurological examinations of both lower limbs were unremarkable. Lumbar radiographs were normal. MRI (Fig. 1) revealed a cystic lesion in the anterior epidural space with low signal intensity on T1-weighted images and high signal intensity on T2-weighted images.

**Fig. 2:**
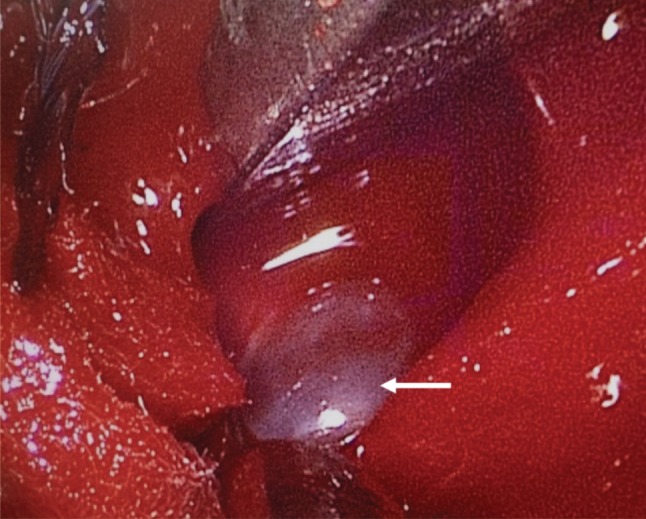
Intraoperative endoscopic image of the discal cyst (indicated by arrow).

The patient underwent endoscopic interlaminar surgery under general anaesthesia, in prone position on Jackson table. The knee and hip were flexed at 90 and 45 degrees, respectively, to increase the space of interlaminar window. The abdomen was left free to avoid increase in intra-abdominal pressure, to reduce venous pooling during the operation. All body prominences were protected and supported with soft silicon gel. Under image intensification, the level to be operated was localised. A 23mm cranio-caudal incision was made over the skin at a junctional point 2/3 lateral to midline and 1/3 medial to medial pedicular line (medial facet). The dorso-lumbar fascia was incised along the plane and widened. The underlying paraspinal muscle was then detached from the spinous process using a periosteal elevator and retracted laterally. The endoscopic portal was then opened followed by the placement of the camera into its respective channel. The working portal was kept flush with the lamina as medial as possible. With a 45-degrees Kerrison rongeur and curette, the ligamentum flavum was incised and detached from the lamina to enable entry into the spinal canal. After the incision of ligamentum flavum and opening of the lamina, the traversing nerve root, thecal sac, and the discal cyst were clearly visualised. The discal cyst was found to be communicating with the L4/L5 intervertebral disc. It (Fig. 2) appeared faintly white in colour, containing serous fluid and was seen to be partially indenting the L5 nerve root. The cyst was excised at the base of the connection by performing an annulotomy. Histopathological examination showed fragment of cyst wall composed of fibrocartilaginous tissue devoid of epithelial lining.

The recovery was uneventful and patient was ambulating well two days after surgery and was discharged home. He had immediate relief of his sciatica symptoms. A week later he was reviewed, and there were neither any nerve root tension signs nor any post-operative complications. At ten months post-surgery, there were no signs indicating any recurrence.

## Discussion

Being a rare clinical entity, discal cyst presents indistinguishably from other causes of lower back pain and radiculopathy. It is an extremely rare pathology with unclear pathogenesis, indeterminate natural history and with no consensus on the ideal management of the condition. An accurate understanding regarding the treatment of the discal cysts remains quite challenging due to limited literature and research in this topic.

The pathogenesis and aetiology of discal cyst still remains unclear despite several proposed hypotheses. Chiba *et al* have proposed a preceding discal injury or disc herniation to have initiated the formation of hematoma as a result of hemorrhage of the epidural venous plexus^1^. On the other hand, Kono *et al* hypothesised that a discal cyst resulted from focal degeneration of an intervertebral disc producing a herniated disc with subsequent spilling of fluid from the herniated disc material that triggered an inflammatory response leading to reactive pseudo-membrane formation that eventually became a discal cyst^4^. Jeong and Bendo supported the theory that the mechanism behind the formation of discal cyst was due to a subsequent change in a herniated disc and not a vascular phenomenon^3^. Based on our intraoperative and histological findings, we agree that the underlying pathology is due to an annular injury leading to a herniated disc with subsequent changes resulting in the formation of a discal cyst.

Discography and CT discography provide a communication channel between the cyst and the corresponding disc, and it is possible to differentiate discal cysts from lumbar disc herniation or other intraspinal cyst^1^,^2^. MRI will demonstrate not only the nature of the cystic lesion, but also its relationship to the thecal sac^3^. It is still controversial whether a discography is necessary in all patients with intraspinal cyst, because irrespective of its origin, surgical removal of the cyst leads to symptom improvement^1^. In our patient, with the aid of MRI and clear surgical indication we proceeded with endoscopic removal of the cyst and intraoperatively confirmed its origin from the adjacent disc.

Operative management of a disc cyst is reserved for patients with persistent neurological symptoms and/or severe leg pain refractory to nonoperative treatment^3^. Nearly all reported discal cysts have been treated surgically^1-5^ and the majority of which underwent open surgery compared to endoscopic surgery. As in our case, we surgically excised the discal cyst via endoscopic interlaminar surgery with immediate relief of symptoms and no postoperative complications. The advantage of this approach is that it directly tackles the pathology in the spinal canal. In contrast to us, Kim *et al* described an interlaminar approach in a lateral decubitus position and the use of endoscope requiring continuous inflow of normal saline^5^. They also utilised a side firing Ho YAG laser for the resection of the cyst.

We agree with Nabeta *et al* that excision as the best treatment for discal cysts in patients with persistent and refractory neurological symptoms and leg pain^2^. We conclude that endoscopic surgery is an effective alternative to conventional open surgery for discal cyst of the lumbar spine.

## Conflict of Interest

The authors declare no conflicts of interest.
